# Anti-Tumor and Anti-Invasive Effects of ONC201 on Ovarian Cancer Cells and a Transgenic Mouse Model of Serous Ovarian Cancer

**DOI:** 10.3389/fonc.2022.789450

**Published:** 2022-03-17

**Authors:** Yali Fan, Jiandong Wang, Ziwei Fang, Stuart R. Pierce, Lindsay West, Allison Staley, Katherine Tucker, Yajie Yin, Wenchuan Sun, Weimin Kong, Varun Prabhu, Joshua E. Allen, Chunxiao Zhou, Victoria L. Bae-Jump

**Affiliations:** ^1^ Department of Gynecologic Oncology, Beijing Obstetrics and Gynecology Hospital, Capital Medical University, Beijing Maternal and Child Health Care Hospital, Beijing, China; ^2^ Division of Gynecologic Oncology, University of North Carolina at Chapel Hill, Chapel Hill, NC, United States; ^3^ Oncoceutics, Philadelphia, PA, United States; ^4^ Lineberger Comprehensive Cancer Center, University of North Carolina at Chapel Hill, Chapel Hill, NC, United States

**Keywords:** ONC201, DRD2, ovarian cancer, invasion, proliferation

## Abstract

ONC201 is a promising first-in-class small molecule that has been reported to have anti-neoplastic activity in various types of cancer through activation of tumor necrosis factor-related apoptosis-inducing ligand (TRAIL) as well as activation of mitochondrial caseinolytic protease P (ClpP). The present study was to explore the anti-tumor potential effect of ONC201 in ovarian cancer cell lines and in a transgenic mouse model of high grade serous ovarian cancer under obese (high fat diet) and lean (low fat diet) conditions. ONC201 significantly suppressed cell proliferation, induced arrest in G1 phase, and increased cellular stress and apoptosis, accompanied by dual inhibition of the AKT/mTOR/S6 and MAPK pathways in OC cells. ONC201 also resulted in inhibition of adhesion and invasion *via* epithelial–mesenchymal transition and reduction of VEGF expression. Pre-treatment with the anti-oxidant, N-acetylcysteine (NAC), reversed the ONC201-induced oxidative stress response, and prevented ONC201-reduced VEGF and cell invasion by regulating epithelial–mesenchymal transition protein expression. Knockdown of ClpP in ovarian cancer cells reduced ONC201 mediated the anti-tumor activity and cellular stress. Diet-induced obesity accelerated ovarian tumor growth in the KpB mouse model. ONC201 significantly suppressed tumor growth, and decreased serum VEGF production in obese and lean mice, leading to a decrease in tumoral expression of Ki-67, VEGF and phosphorylation of p42/44 and S6 and an increase in ClpP and DRD5, as assessed by immunohistochemistry. These results suggest that ONC201 may be a promising therapeutic agent to be explored in future clinical trials in high-grade serous ovarian cancer.

## Introduction

Ovarian cancer (OC) is the second most common gynecological cancer and the most lethal gynecologic malignancy in the United States, with an estimated 21,410 new cancer cases and 13,770 cancer deaths in 2021 (1). At least 70% of patients are diagnosed at advanced stages, and no specific symptoms or diagnostic tools are currently available for early diagnosis (2). Despite treatment with aggressive debulking surgery and combination chemotherapy (carboplatin and paclitaxel), up to 75% of patients with advanced OC experience tumor progression or recurrence, with a dismal 5-year overall survival (OS) of 25%, largely due to the emergence of drug resistance (2, 3). Therefore, the identification of novel target molecules and the development of new therapeutic agents are desperately needed to improve outcomes for this highly lethal disease.

ONC201 is a selective competitive and noncompetitive antagonist of the G protein coupled receptor (GPCR) dopamine receptor D2 (DRD2) that was identified in a high-throughput phenotypic cell-based screen as an efficacious anti-tumorigenic therapeutic agent (4). Anti-tumorigenic mechanisms of ONC201 stems from stimulation of activating transcription factor 4 (ATF4) as well as the C/EBP homology protein (CHOP)-mediated integrated stress response (ISR), ultimately leading to induction of death receptor 5 (DR5) and tumor necrosis factor-related apoptosis-inducing ligand (TRAIL) and inhibition of Akt/ERK signaling pathways (4, 5). Dopamine receptor D5 (DRD5) acts as a direct negative regulator of DRD2 signaling pathway to regulate the anti-tumor effects of ONC201 (6). More recent studies found that mitochondrial caseinolytic protease P (ClpP) is another critical target for ONC201, and activation of ClpP by ONC201 results in modulation of the ISR pathway, inhibition of protein synthesis and induction of mitochondrial dysfunction, which eventually leads to inhibition of tumor growth *in vitro* and *in vivo* (7, 8). Pre-clinical studies have shown that ONC201 presents anti-tumorigenic effects in dopamine pathway-dysregulated solid tumors and hematologic malignancies (5, 9, 10). We recently found that ONC201 exhibited anti-tumorigenic and anti-metastatic activity in uterine serous carcinoma (USC) *in vitro* and inhibited tumor growth in a transgenic mouse model of endometrial cancer under obese and lean conditions (11, 12). Phase I clinical trials revealed that ONC201 is clinically active and exceptionally well-tolerated with favorable pharmacokinetics and pharmacodynamics in a variety of cancers, including lymphoma, glioblastoma and endometrial cancer (4, 11, 13–16). Currently, multiple phase II clinical trials are underway to evaluate the single agent therapeutic efficacy of ONC201, including trials in endometrial and OC (4, 17).

Given that ONC201 has unique features including broad-spectrum activity independent of mutations or tumor type, oral administration and excellent safety profile, our objective was to evaluate the effect of ONC201 on cell proliferation, cellular stress, apoptosis, invasion and tumor growth in OC cell lines and a genetically engineered mouse model of high grade serous OC. Additionally, given that obesity may be associated with OC risk and adverse survival in patients with OC, and there is inter-relationship between mitochondrial dysfunction and obesity as well as the effect of ONC201 on the mitochondrial regulator ClpP, we also studied the anti-tumorigenic effects of ONC201 in both obese and lean mice with OC. Our results show that ONC201 demonstrates promise as a single targeted agent for OC, worthy of further evaluation in clinical trials.

## Methods

### Cell Culture and Reagents

Four ovarian cancer cell lines, OVCAR3, OVCAR5, IGROV-1 and SKOV3, were used in this study. The cells were grown in DMEM/F12(1:1) supplemented with 10% fetal bovine serum, 2 mM L-glutamine, 100U/ml penicillin and 100 ug/ml streptomycin under 5% CO_2_. ONC201 was kindly provided by Oncoceutics, Inc. MTT, propidium iodide and RIPA buffer were purchased from Sigma (St. Louis, MO). Antibodies to PERK (#5683), ATF4 (#11815), CHOP (#2895), BCL-XL (#2764), MCL-1 (#5453), PARP (#9542), DR5 (#8074), β-actin (#3722), VEGF-C (#2445), Slug (#9585), Snail (#3879), CyclinD1 (#2978), CDK4 (#12790), and CDK6 (#3136) were obtained from Cell Signaling Technology (Beverly, MA). DRD2 (B-10, sc-5303) and DRD5 (E-12, sc-376088) were purchased from Santa Cruz Biotechnology. The TMRE (#22220) and JC-1 (#22200) probes were purchased from AAT bioquest (Sunnyvale, CA).

### Cell Proliferation Assay

The four OC cell lines were incubated in 96-well plates (4000 cells/well) in the presence of varying concentrations of ONC201 for 72 hours. The control group received vehicle only (0.1% DMSO). 5ul MTT (5 mg/mL) was added in each well following by incubation for 1-2 hours at 37°C. MTT absorbance was measured at 575 nm after 100 ul DMSO was added to the plates. Effects of ONC201 on cell proliferation were assessed as a percentage of control cell proliferation obtained from 0.1% DMSO treated cells grown in the same 96-well plates followed by IC50 analysis.

### Colony Formation Assay

The OVCAR5 and SKOV3 cells were plated at a density of 200 cells in 6-well plates in triplicated for each treatment group for 24 hours. The cells were treated with indicated concentrations of ONC201 or vehicle controls for 48 hours and then incubated at 37°C for 10-14 days. The culture medium was changed every third or fourth day. The cells were fixed in methanol and stained with 0.5% crystal violet. The colonies were counted utilizing Photoshop software.

### Cell Cycle Analysis

The cells were grown in in 6-well plates overnight and then incubated with varying concentrations of ONC201 or vehicle control for 36 hours. Cells were harvested, washed with PBS, fixed in 2 ml of ice-cold 90% ethanol and stored overnight at -20°C until cell cycle analysis was performed. On the day of analysis, the cells were resuspended in 100 ul RNase A solution containing propidium iodide (2 mg/ml) for 30 min at room temperature in the dark. DNA content was determined by Cellometer (Nexcelom, Lawrence, MA). Cell cycle was analyzed using FCS4 express software (Molecular Devices, Sunnyvale, CA).

### Annexin V Assay

Cell apoptosis was determined using Annexin-V FITC kit (BioVision, Mountain View, CA) following manufacture’ protocol. The cells were seeded into 6-well plates at a density of 2.5 × 10^5^ cells/well and then treated with vehicle or varying concentrations of ONC201 for 30 hours. The cells were harvested and stained with 100 ul of Annexin-V and PI dual-stain solution for 15 min in the dark. Annexin V expression was determined by a Cellometer. Apoptosis cells were analyzed by FCS4 express software.

### Caspase 3, 8 and 9 Assays

Caspase activity assays were performed with modifications as previously described (18). In brief, the OVCAR5 and SKOV3 cells were seeded in 6-well plates at a density of 2.5 × 10^5^ cells/well overnight. The cells were treated with ONC201 at different concentrations for 8-12 hours. The controls cells are treated with cell culture media at DMSO concentration of 0.1%. 150-180 ul 1X caspase lysis buffer was added to each well. BCA protein assay was used for quantitation of protein concentration. 10-15 ug lysates in a black clear bottom 96-well plate were incubated with reaction buffer and 200 uM of caspase substrates for 30 min. The fluorescence of each well was determined using a microplate reader (Tecan, Morrisville, NC). The selective substrates Ac-DEVD-AMC, Ac-IETD-AFC and Z-IETD-AFC (AAT Bioquest) were used for caspase 3, caspase 8 and caspase 9, respectively. Each experiment was repeated three times to assess for consistency of results.

### Reactive Oxygen Species (ROS) Assay

ROS production was determined using the DCFH-DA assay, as previously described (19). The OVCAR5 and SKOV3 cells (8000 cells/well) were cultured in a black 96-well plate overnight followed by treatment with indicated doses of ONC201 or vehicle for 12 hours. Cells were then incubated with 20 uM DCFH-DA in regular growth medium for 30 minutes. ROS accumulation was measured by a plate reader (Tecan) at an excitation wavelength of 485 nm and an emission wavelength of 530 nm.

### Mitochondrial Membrane Potential Assay

Mitochondrial membrane potential was analyzed using the specific fluorescent probes JC-1 and TMRE, respectively (20, 21). The cells were cultured overnight in a 96 well plate and then treated with different concentrations of ONC201 or vehicle control for 8 hours. Treated cells were incubated with 2 uM JC-1 or 1 uM TMRE for 30 minutes at 37°C. The levels of the fluorescent probes were measured using a Tecan plate reader at Ex/Em= 549/575 nm for TMRE. For JC-1, green JC-1 signals were measured at Ex/Em 485/535 nm and red signals were measured at 535/590 nm. Each experiment was repeated three times to assess for consistency of results.

### Adhesion Assay

Cell adhesion was measured by using laminin adhesion assay as previously described (11). Briefly, a 96-well plate was coated with 100 ul laminin-1 (10 ug/ml) for 1 hour and was blocked by 3% BSA for 30 min at 37°C. The cells were pre-treated with ONC201 for 24 hours and then added to the laminin coated wells (25,000 cells). The plate was incubated at 37°C for 2 hour in serum-free medium. The cells were washed twice with PBS to remove nonadherent cells. Attached cells were fixed by adding 100 ul of 5% glutaraldehyde for 20-30 min and stained with 0.1% crystal violet for 30 min. Next, the cells were lysed with 10% acetic acid and the absorbance of the solution was measured at 570 nm in a plate reader (Tecan).

### Transwell Assay

Cell invasion assays were performed using a 96-well plate coated with 0.5-1 x BME. The OVCAR5 and SKOV3 cells were starved for 12 hours and then seeded in the upper chambers of the wells and the lower chambers were filled with regular medium and differing concentrations of ONC201. The media contained 0.1% DMSO as a vehicle control. The plates were incubated for 4 hours to allow invasion into the lower chambers. After washing the upper and lower chambers with PBS, 100 ul of calcein AM solution (Invitrogen, Carlsbad, CA) was added to the lower chambers and incubated for 30-60 minutes. The lower chamber plate was measured by plate reader for reading fluorescence at EX/EM 485/520 nm. Each experiment was repeated twice.

### Wound Healing Assay

The cells were plated in 6-well plates at 3.5 × 10^5^ cells/well for 24 hours and then replaced with media with 0.5% charcoal stripped FBS for 12 hours. A sterilized 200 ul pipette tip was used to draw a straight line across the plate in one direction. The cells were then washed with fresh media to remove the detached cells, and cells were then treated with different concentrations of ONC201 in the media supplemented with 0.5% charcoal stripped serum for 24 to 48 hours. The images were acquired at different timing points (24, 36 and 48 hours). Measurements of the width of the wound were performed at random intervals with the Adobe Photoshop CS6.

### Organotypic 3D Co-Cultures

The organotypic culture was performed as previously described (22). 1 × 10^6^ mouse stromal cells transfected with hTERT were mixed with 3.5 volumes of Matrigel^®^, 1 volume of 10X DMEM, 1 volume of FCS, and 1 volume of DMEM/F12, and then were pipetted into each well of a 24-well plate. After 18 hours, 5 × 10^5^ OVCAR5 cells, suspended in 1 ml regular media supplemented with 10% FCS, were added to each well. The OVCAR5 cells were treated with 10 uM ONC201 or vehicle control for 36 hours. Then the gel with stromal and cancer cells was lifted and placed onto a stainless steel grid in a 6 well plate. DMEM/F12 media was added to the well so that the gel plug was exposed to the air from above and to the media from below. The media was changed every 3 days maintaining the air-liquid interface. The organotypic gels were cultured for 12 to 14 days and then fixed in 4% formaldehyde for 24 hours. The gels were bisected and processed to paraffin blocks. Each slide was stained using standard haematoxylin and eosin (H&E), and then scanned by Motic. The “Invasion Index” was calculated by ImagePro as MCY × N × A (MCY: Mean Cord Y, N: the number of cancer islands, A: sum of areas of the cancer islands). The gels not treated with ONC201 were used as control.

### Transient ClpP Knockdown

The OVCAR5 and SKOV3 cells were transiently transfected with either siRNA targeting ClpP (15 nM) and scrambled control siRNA (15 nM) using Mission SiRNA Transfection Reagent (Sigma, St. Louis, MO), according to manufacturer’s protocol. MTT assay were performed at 48 hours post-transfection. Western blotting were performed at 24 hours after transfection.

### Western Immunoblotting

The OVCAR5 and SKOV3 cells were treated with ONC201 or vehicle for 30 hours. Total cell lysates were prepared in RIPA buffer plus PhosStop. Protein concentration was quantified using BCA assay (Sigma, St. Louis, MO). Equal amounts of lysates were electrophoresed on 10-12% SDS-PAGE and transferred onto a PVDF membrane. The membranes were probed at 4°C overnight with appropriate primary antibodies. Proteins were visualized using SuperSignal West Pico Substrate (Thermo Scientific, Waltham, MA) by the ChemiDoc image system (Bio Rad, Hercules, CA).

### ONC201 Treatment in KpB Mouse Model

The KpB ( $\text{K}18{\text{-gT}}_{121}^{+/-}$ ;p53^fl/fl^;Brca1^fl/fl;^ KpB) transgenic mouse model of high grade serous epithelial OC has been described previously in detail (23–25). Animal protocol was approved by the UNC-CH Institutional Animal Care and Use Committee (IACUC). Mice were housed on a 12 hours light, 12 hours dark cycle, with free access to food and water The KpB female mice were fed a high fat diet (HFD, 60% calories from fat) or a low fat diet (LFD, 10% calories from fat, Research Diets) at 3 weeks of age. 5 ul of 2.5 x10^7^ P.F.U of recombinant adenovirus Ad5-CMV-Cre (AdCre, Transfer Vector Core, University of Iowa) was injected into the left ovarian bursa cavity at 6–8 weeks age (24). The mice were checked weekly by abdominal palpation for the appearance of ovarian tumors. Once tumors had reached an average size of 0.1x0.1 cm in diameter by palpation, the mice fed with HFD or LFD were assigned into the four groups: HFD control, LFD control, HFD+ONC201 and LFD+ONC201 (N=15/group). ONC201 was given weekly in 0.1 ml containing either 130 mg/kg or placebo in oral gavage for 4 weeks. The size of ovarian tumors were measured twice a week using palpation. The mice were weighted weekly and observed daily any signs of toxicity or distress. No mice died during the treatment. After 4 weeks of treatment, mice were sacrificed by CO2 asphyxiation and tumors were weighted. Ovarian tumor volumes were calculated as following: (width2 × length)/2.

### Immunohistochemical Analysis

Five micrometer paraffin sections from the KpB mice tumors were processed for IHC analysis at IHC Mice Core Facility at UNC. Briefly, the slides were incubated with Ki-67, phosphorylated p42/44, phosphorylated-S6, and VEGF at 4°C overnight, respectively, and then treated with HRP-conjugated antibody for 1 hour. The sections were further applied with ABC-Staining Kits (Vector Labs, Burlingame, CA) for color reaction and hematoxylin for counterstaining. All IHC slides were scanned by Motic and scored by ImagePro software (Rockville, MD).

### Serum VEGF Assay

The VEGF productions of mice serum after treatment with ONC201 were detected using a VEGF ELISA Kit (#MMV00, R&D Systems, Minneapolis, MN), according to the manufacturer’s directions. Each sample from ONC201 and control groups was measured in duplicate. Plates were read at 570 nm using a Tecan plate reader.

### Statistical Analysis

Data are given as the mean ± SD. Statistical significance was analyzed by the two-sided unpaired Student’s t-test from at least three replicates. Tumor growth in different treatment arms was analyzed by One-way & Two-way ANOVA test. GraphPad Prism 5 (La Jolla, CA USA) was used for all graphs and significance tests. P values of <0.05 were considered to have significant group differences.

## Results

### ONC201 Inhibited Cell Viability in OC Cell Lines

**Figure 1:**
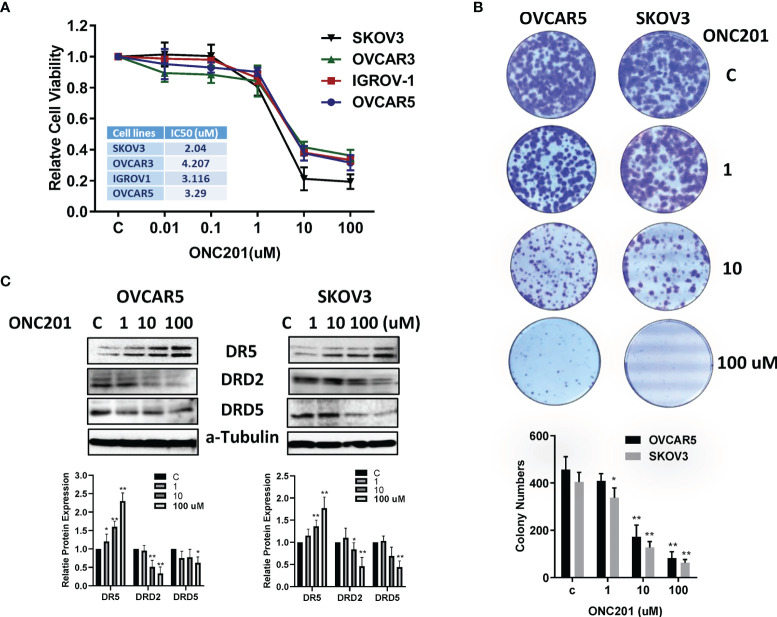
ONC201 inhibited cell viability and colony formation. The OVCAR5, OVCAR3, IGOV-1 and SKOV3 in 96 well plates were treated with increasing concentrations of ONC201 for 72 hours and subjected to the MTT assay. ONC201 significantly inhibited cell growth in a dose- dependent manner in all cell lines. Similar results were obtained from three independent experiments **(A)**. ONC201 inhibited colony forming ability of OVCAR5 and SKOV3 cells compared to the vehicle-treated cells. Cells were incubated with ONC201 for 48 hours, then cell were culture in drug-free media for 12 days **(B)**. The images and bar chart represented results of one experiment. Western Blotting was used to evaluate the effect of ONC201 on expression of DRD2, DRD5 and DR5 in the OVCAR5 and SKOV3 cells. ONC201 decreased the expression of DRD2 and DRD5 and increased the expression of DR5 in a dose-dependent manner in both cell lines **(C)**. * <0.05, ** <0.01.

ONC201−mediated inhibition of OC cell viability was assessed using MTT assay. The OVCAR3, IGROV-1, OVCAR5 and SKOV3 were cultured in media with various concentrations of ONC201 for 72 hours. The MTT results showed that with increasing ONC201 concentrations, a dose-dependent growth inhibition was observed in four OC cell lines compared to the control cells (**Figure 1A**). The mean IC50 values of ONC201 were 4.2 uM for OVCAR3, 3.1 uM for IGROV-1, 3.2 uM for OVCAR5 and 2.1 uM for SKOV3, respectively. Subsequently, because colony formation assay is a well-established *in vitro* technique for testing the proliferative capability of treated cells (26), we investigated the long-term effect of ONC201 on OC cell growth. OVCAR5 and SKOV3 cells were seeded in same density in six-well plates and incubated 1, 10 and 100 uM of ONC201 for 2 days and subsequent culture of the cells for 12 days. The results showed that the colony-forming ability was reduced by 4.8%, 58.3% and 79.75% in OVCAR5, and 22.2%, 57.5% and 86.1% in SKOV3, respectively, after the cells were treated with 1-100 uM ONC201 compared with vehicle control (**Figure 1B**). These results suggest that OC cells are sensitive to the anti-proliferative effects of ONC201.

Because ONC201 is a selective antagonist of the G protein-coupled receptor DRD2 that causes p53-independent apoptosis through upregulation of TRAIL and DR5 in tumor cells (4), we next detected the effect of ONC201 on DRD2, DRD5 and DR5 in the OVCAR5 and SKOV3 cells. The cells were treated with ONC201 at 1, 10 and 100 uM for 24 hours. Western blotting results showed that Treatment with OVCAR5 and SKOV3 cells with 10 or 100 uM ONC201 reduced the protein levels of DRD2 and DRD5, with DR5 expression being increased compared with vehicle control (**Figure 1C**).

### ONC201 Caused Cell Cycle G1 Arrest in OC Cells

**Figure 2:**
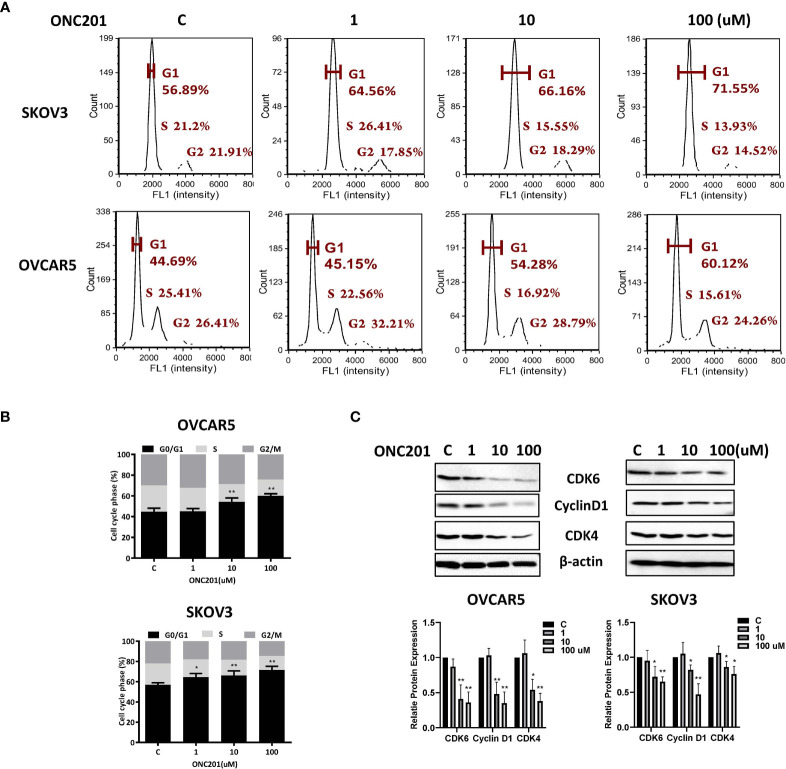
Induction of cell cycle G1 arrest by ONC201 is dose-dependent. The OVCAR5 and SKOV3 cells were incubated with indicated doses of ONC201 for 36 hours. Cell cycle progression was assessed by Cellometer. Cell cycle G1 arrest was found in both cell lines in a dose-dependent manner **(A, B)**. The cells were exposed to 1-100 um ONC201 or the vehicle controls for 36 hours prior to western blotting for the detection CDK4, CDK6 and Cyclin D1. ONC201 decreased the expression of CDK4, CDK6 and Cyclin D1 in both cell lines. β-actin was used as loading control **(C)**. The results shown are one of three independent experiments. * <0.05, ** <0.01.

We next investigated whether ONC201 modulates cell cycle progression in OC cells. As illustrated in **Figures 2A, B**, treatment with 1, 10 and 100 uM ONC201 for 36 hours caused significant increases in the G1 phase and decreases S phase in a dose-dependent manner in the OVCAR5 and SKOV3 cells. G1 arrest phase increased from 44.69% in control cells to 60.12% in the 100 uM ONC201-treated OVCAR5 cells and 56.89 to 71.55% in the SKOV3 cells; in parallel, the S phase cell population decreased from 25.41 to 15.62% with increasing concentrations of ONC201 in the OVCAR5 and 21.2 to 13.93% in the SKOV3 cells (p<0.05), respectively. To further understanding the mechanism underlying the cell cycle arrest, cell cycle-related proteins were analyzed by western blotting in the ONC201-treated OVCAR5 and SKOV3 cells. The results showed that ONC201 resulted in reduced expression of cyclin D1 in both cell lines after 36 hours of treatment. Moreover, ONC201 also inhibited the expression of the cyclin D1 regulatory partners, CDK4 and CDK6, following ONC201 treatment for 36 hours compared with vehicle control (**Figure 2C**). These data suggest that ONC201 induces cell cycle G1 arrest through cyclin D1 degradation in OC cells.

### ONC201 Induced Apoptosis in OC Cells

**Figure 3:**
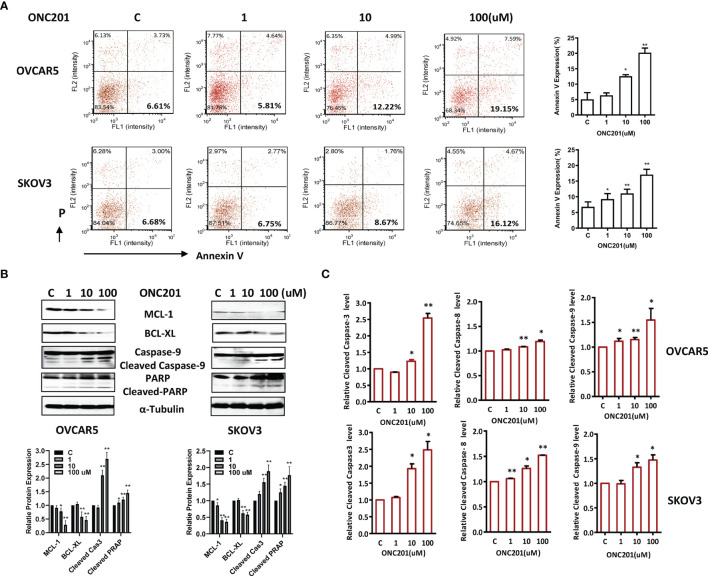
ONC201 induced apoptosis in OC cells. OVCAR5 and SKOV3 cells were incubated for 30 hours with the indicated amount of ONC201. Annexin V was analyzed by Cellometer **(A)**. Total cell lysates were prepared and analyzed by western blotting for MCL-1, BCL-XL, Cleaved caspase 9 and PARP **(B)**. Cleaved caspase 3, 8 and 9 activity was assayed by ELISA assay. ONC201 induced the activity of the cleaved caspases 3, 8 and 9 in both cell lines after treatment with ONC201 or the vehicle controls for 8-12 hours **(C)**. The results are shown as the mean ± SD and are representative of three independent experiments. *p < 0.05, **p < 0.01.

To characterize the underlying mechanism of growth inhibition by ONC201, the apoptotic cells were analyzed by performing an Annexin-V assay after treating the OVCAR5 and SKOV3 cell lines with ONC201 (1-100 uM). Annexin-V analysis showed a meaningful increase in apoptotic cells in a dose-dependent manner after 30 hours of ONC201 treatment, along with decreasing expression of MCL-1 and BCL-XL protein (**Figure 3A**). Annexin V expression increased from 6.61% in control cells to 19.15% in the 100uM ONC201-treated OVCAR5 cells and 6.68 to 16.12% in the SKOV3 cells, respectively (p<0.01). To examine whether ONC201 induces apoptosis through the mitochondrial apoptosis pathways in the OC cells, western blotting results showed that ONC201 significantly induced cleaved PARP and caspase 9 protein expressions in both cell lines compared with vehicle control (**Figure 3B**). Furthermore, a dose-dependent increase in the activity of cleaved caspase 3, 8 and 9 was found by ELISA assays in OVCAR5 and SKOV3 cells in response to ONC201 treatment (**Figure 3C**). These results indicated that apoptosis induced by ONC201 was executed through activation of either the extrinsic pathway (death receptor) or the intrinsic pathway in OC cells.

### ONC201 Inhibited Tumor Growth in a Transgenic Mouse Model of OC

**Figure 4:**
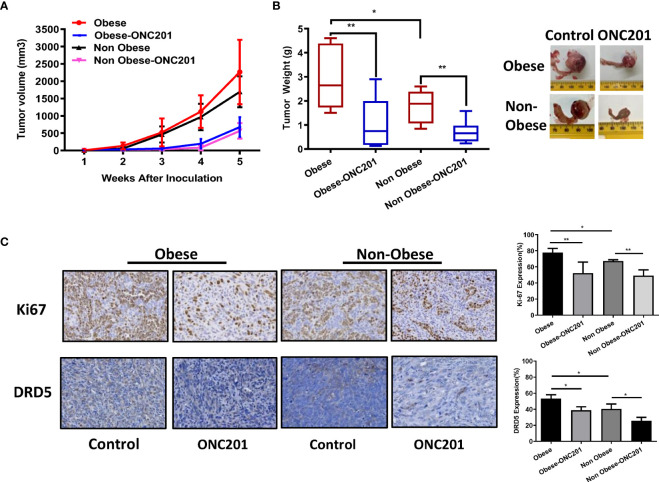
ONC201 inhibited tumor growth in KpB mouse model of OC. Obese or lean KpB mice were treated with ONC201 (130 mg/kg, weekly) or vehicle for 4 weeks. Tumor volumes of ovarian tumors were measured weekly. The mean tumor volume was reduced in obese or lean mice treated with ONC201 **(A)**. Tumor weight measurements of KpB mice were recorded at the time of sacrifice **(B)**. The expression of DRD5 and Ki67 was assessed by immunohistochemistry analysis in ovarian tumors following ONC201 treatment. The results showed that ONC201 decreased the expression of Ki-67 and DRD5 in the ovarian tumor tissues under obese and lean conditions **(C)**. *p < 0.05; **p < 0.01.

Given that obesity is associated with worse outcomes for ovarian cancer and dopamine signaling pathway contributes to the distinct metabolic profiles of obese and non-obese patients, we sought to evaluate whether ONC201 was able to inhibit tumor growth in a transgenic mouse model of high grade serous OC (KpB) under obese and non-obese conditions. Immune competent mice carrying Cre-inducible oncogenic Brca1 in combination with deletion of p53 and Rb in the ovaries develop high grade serous OC about 4-6 months after injection with AdCre (25). The HFD-fed or LFD-fed KpB mice were divided into four groups (15 mice/group): HFD, HFD+ONC201, LFD and LFD+ONC201, respectively. The mice were treated once a week by oral gavage with either ONC201 (130mg/kg, 4 weeks) or placebo after tumor induction. The tumor size was monitored twice a week by palpation. The mice showed tolerance to ONC201 treatment and did not show any obvious changes in behavior and body weight. After 4 weeks of treatment, the tumors were excised, weighed, and examined histologically. ONC201 effectively inhibited tumor growth and reduced tumor weight in both HFD and LFD groups compared to control groups. Ovarian tumor weights in obese KpB mice were significantly greater than that in the non-obese control mice (2.92 g versus 1.80 g, p<0.05), suggesting that obesity promoted ovarian tumor growth. ONC201 treatment decreased tumor weight by 75.5% in obese mice and 65.2% in non-obese mice compared to their control groups (**Figures 4A, B**, p<0.01). These findings imply that ONC201 caused similar anti-tumor effects in obese mice compared to non-obese KpB mice although gene expression and metabolomics profiling showed statistically significant differences between the ovarian tumors from the obese versus lean mice (27). Collectively, these data demonstrate that treatment with ONC201 significantly suppressed OC growth in a genetically engineered mouse model of OC under obese and non-obese conditions.

To determine the anti-tumorigenic mechanisms of ONC201 *in vivo*, the expression of Ki-67 and DRD5 in ovarian tumors was evaluated by IHC in obese and non-obese KpB mice after 4 weeks of treatment. The tumors from obese mice displayed increased expression of the Ki67 and DRD5 compared to non-obese mice (p<0.05). Consistent with our results *in vitro*, ONC201 inhibited Ki-67 expression in the ONC201-treated obese mice by 33.6% compared with obese control mice and the ONC201-treated lean mice by 26.1% compared with lean control mice (p<0.01). In addition, we found that high expression of DRD5 was identified in ovarian tumors of obese mice, and the expression of DRD5 was reduced in the obese and lean mice treated with ONC201 compared with the control mice (**Figure 4C**, p<0.05), suggesting that ONC201 inhibits ovarian tumor growth through the DRD2/DRD5 pathway.

### ONC201 Induced Cellular Stress in OC Cells

**Figure 5:**
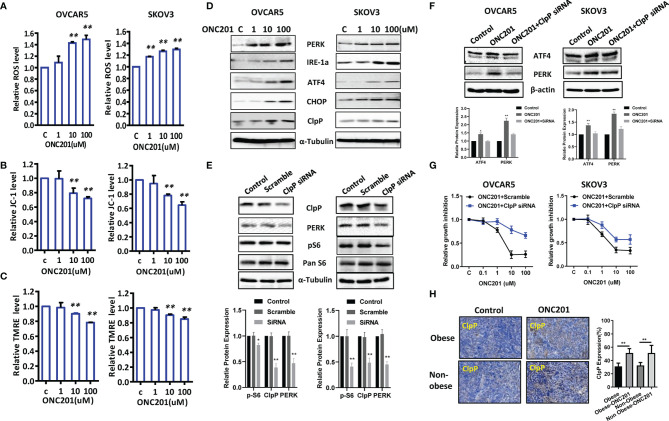
ONC201 induced ER stress in OC cells. Cell stress analysis was done on OVCAR5 and SKOV3 cells treated with ONC201. ROS, JC-1 and TMRE products were measured by ELISA assays. ONC201 significantly increased the levels of ROS and decreased mitochondrial membrane potential in both cell lines compared to the vehicle -treated cells **(A-C)**. ONC201 increased the expression of cellular stress related proteins including PERK, IRE-1a, ATF4, CHOP and ClpP in both cell lines after 24 hours of treatment **(D)**. Knockdown ClpP reduced the expression of PERK and phosphorylated S6 in both cells **(E)**. ClpP siRNA decreased ONC201 mediated ATF4 and PERK expression and ONC201 induced cell inhibition in both cells **(F, G)**. In KpB mice, ONC201 also induced ClpP expression in the ovarian tumors under obese and lean conditions, as measured by IHC **(H)**. * <0.05, ** <0.01.

ROS have been implicated as mediators of TRAIL-induced apoptosis in cancer cells *via* different pathways (28). To examine the involvement of oxidative stress in the anti-tumorigenic effect of ONC201 in OC cells, intracellular ROS levels were detected using the DCFH-DA assay. The results showed an increase in ROS production when OVCAR5 and SKOV3 cells were treated with ONC201 at different concentrations for 12 hours. At a concentration of 100 uM, ONC201 significantly increased DCFH-DA fluorescence 1.49 and 1.30- fold in OVCAR5 and SKOV3 cells compared with vehicle control cells (p<0.01), respectively (**Figure 5A**).

To further evaluate the underlying mechanism of ROS effect in association with mitochondrial function, we next set out to assess the ability of ONC201 to depolarize mitochondrial membranes by JC-1 and TMRE ELISA assays. JC-1 assay indicated that ONC201 induced the loss of mitochondrial transmembrane potential (ΔΨm) in both cell lines after 8 hours of treatment compared to control cells. ONC201 at 10 uM significantly reduced ΔΨm by 20.8% and 23.5% in the OVCAR5 and SKOV3 cells compared with vehicle control (p<0.01), respectively (**Figure 5B**). Similarly, these changes of ΔΨm in response to treatment were also observed in a TMRE assay in both cell lines (**Figure 5C**), which further strengthens the reliability of our results. Moreover, western blotting analysis showed that ONC201 significantly increased expression of mitochondrial protease, ClpP, and endoplasmic reticulum (ER) stress-related markers including ATF4, PERK, CHOP and IRE-1a in a dose-dependent manner in both cell lines after 24 hours of treatment (**Figure 5D**).

ClpP is essential for the oxidative stress response, and ONC201 is an allosteric agonist of ClpP. To understand the effect of ClpP on ONC201 mediated cell proliferation and cell stress, OVCAR5 and SKOV3 cells were transfected with ClpP siRNA or scramble RNA, respectively. As shown in **Figure 5E**, ClpP siRNA significantly downregulated the protein expression of ClpP in both cells compared with cells transfected with mock siRNA, which was accompanied by a decrease in PERK expression in both cells. Knockdown of ClpP resulted in decreased expression of phosphorylated S6 in SKOV3 cells. However, in OVCAR5 cells, this effect on phosphorylated S6 was not as pronounced as in SKOV3 cells. The siRNA-mediated knockdown of ClpP effectively inhibited the expression of ATF4 and PERK induced by ONC201 in OVCAR5 cells (**Figure 5F**). ONC201 mediated inhibition of cell proliferation in OVCAR5 and SKOV3 cells partially recovered by transfection with ClpP siRNA (**Figure 5G**). Importantly, IHC results confirmed ONC201 significantly increased the expression of ClpP in obese and lean mice after 4 weeks of treatment (**Figure 5H**). These results suggest that activation of ClpP through ONC201 is involved in its anti-tumorigenic activity in OC.

### ONC201 Inhibited Adhesion and Invasion in OC Cells and Ovarian Tumors

**Figure 6:**
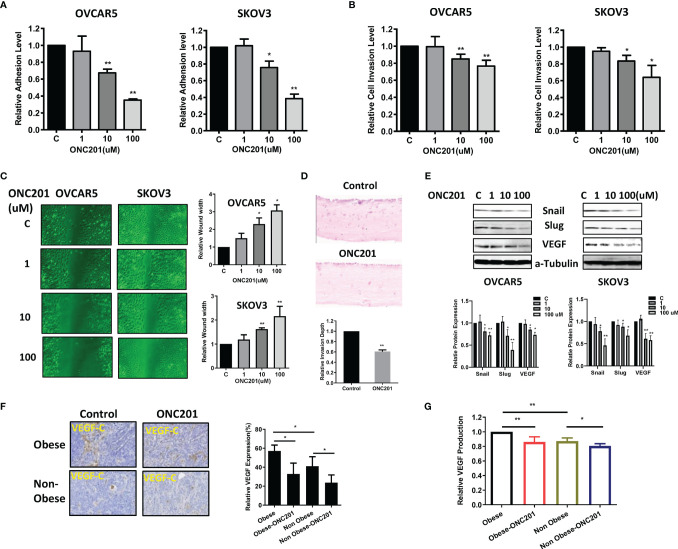
ONC201 inhibited adhesion and invasion. The OVCAR5 and SKOV3 cells were treated with ONC201 at a range of doses from 1−100 μM. laminin-1 assay was used to assess adhesion after 2 hour of treatment with ONC201**(A)**. Invasion was examined by transwell assay after 4 hours of treatment with ONC201**(B)**. Treatment of both cells with 10 and 100 uM ONC201 inhibited cell adhesion and invasion. Migration was assessed by wound healing assay after treatment with ONC201 for 48 hours. ONC201 (10 and 100 uM) significantly reduced cell migration in both cell lines **(C)**. Organotypic 3D co-culture using OVCAR5 cells with and without ONC201 (10 uM) was carried out and the relative invasion depth calculated. ONC201 decreased the invasion index in OVCAR5 cells **(D)**. Western blotting found that ONC201 reduced the expression of VEGF, Snail and Slug in both cell lines **(E)**. The obese and lean KpB mice were treated with ONC201 for 4 weeks. IHC results showed that ONC201 reduced VEGF expression in both obese and lean mice **(F)**. Obesity was associated with increased serum VEGF production as compared to lean mice, and ONC201 reduced the production of VEGF in serum under obese and lean conditions **(G)**. * <0.05, ** <0.01.

Given that ONC201 exhibited anti-invasive ability in USC cells (11), we investigated the impact of ONC201 on cell adhesion, migration and invasiveness in the OVCAR5 and SKOV3 cell lines. In the assessment of cell adhesion, both cell lines were incubated in laminin-coated 96 well plates and treated with ONC201 for 2 hours. As shown in **Figure 6A**, cellular adhesion was decreased by 61.6% to 35.1% in the SKOV3 and OVCAR5 cells, respectively, at a dose of 100 uM compared with control cells (p<0.01). Cell invasion was measured using a transwell invasion assay with a Matrigel-coated filter. Both cell lines were seeded in the upper chambers of the transwell and treated with ONC201 (1-100 uM) for 4 hours. The invasive capacity of the OVCAR5 and SKOV3 cell lines was reduced by ONC201 treatment in a dose-dependent manner. ONC201 (100 uM) significantly reduced the invasive ability of the OVCAR5 and SKOV3 cell lines by 23.3% and 36.0% compared with vehicle control cells (**Figure 6B**, p<0.05).

To evaluate the effect of ONC201 on cell migration, a wound-healing assay was performed in OVCAR5 and SKOV3 cells. The cells were treated with different concentrations of ONC201 and evaluated the cell migration capacity at 24, 48 and 72 hours. ONC201 had a significant inhibitory effect on cell migration of OVCAR5 and SKOV3 cells at all times compared with vehicle-treated control cells (p<0.05), which was most pronounced at 48 hours of treatment as shown in **Figure 6C**.

Given that stromal cells have an active role in inducing EMT and enhancing invasive potential, and the pattern of invasion produced in organotypic cultures displays a similar invasion patterns observed in human (29), we used organotypic cultures containing stromal cells to measure the invasion capacity of OC cells after treatment with ONC201. The organotypic gels stained with H&E were analyzed to generate an invasion index (30). Similar to wound healing and transwell assays, 14 days after treatment, addition of 10 uM ONC201 for 24 hours significantly reduced the invasion index, and OVCAR5 cells invasion was inhibited by 39.3% in organotypic cultures compared to control (**Figure 6D**, p<0.01).

We next examined the effect of ONC201 on the EMT and angiogenesis in the OC cells. Treatment with ONC201 for 24 hours significantly decreased the expression of VEGF-C, Slug and Snail in both cell lines (**Figure 6E**). IHC results showed that obesity increased VEGF expression in the ovarian tumors, when comparing tumors from obese versus lean mice. However, ONC201 reduced VEGF expression by 42.5% in the obese mice and by 42.2% in the lean mice as compared to controls (**Figure 6F**, p<0.05). In addition, we found that the production of serum VEGF decreased by 14.0% in obese mice and 19.4% in lean mice in comparison to placebo-treated mice (**Figure 6G**). Together, these findings support the contention that ONC201 has an ability to inhibit adhesion, invasion and angiogenesis in OC cells and ovarian tumors in KpB mice.

### Inhibition of Cellular Stress Reduced the Effects of ONC201 on Cell Proliferation and Invasion

**Figure 7:**
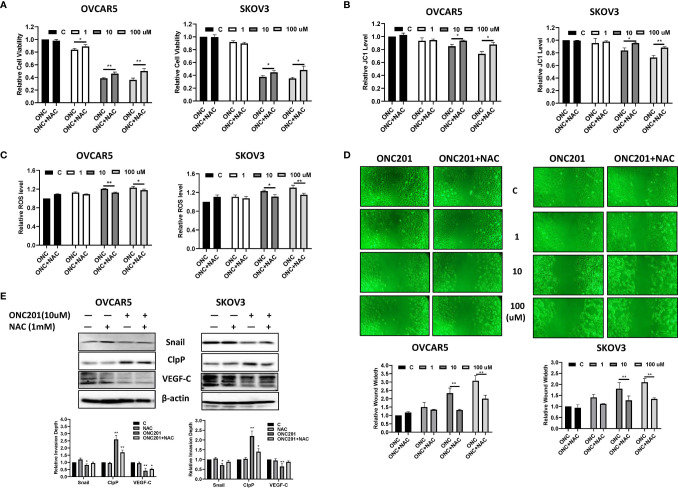
ONC201 inhibited adhesion and invasion through cellular stress pathways in OC cells. Pre-treatment of the OVCAR5 and SKOV3 cells with 1 mM NAC partially blocked inhibition of ONC201-mediated proliferation in both cell lines **(A)**. Similarly, pre-treatment with NCA for 6 hours in OVCAR5 and SKOV3 cells significantly reduced ROS levels and recovered mitochondrial membrane potential induced by ONC201 compared to the vehicle controls **(B, C)**. The cells were pre-treated with NAC for 6 hours, followed by treatment with ONC201 for 48 hr. Wound healing was observed by a microscope with phase contrast. ONC201 decreased cell migration, and NAC effectively reversed ONC201-inhibited cell migration in the OVCAR5 and SKOV3 cells. All images were obtained from three independent experiments **(D)**. The effect of NAC and ONC201 on the expression of ClpP, VEGF and Snail was examined by Western blotting in OVCAR5 and SKOV3 cell lines after 30 hours of treatment **(E)**. *P < 0.05, **P < 0.01.

Because ONC201 is a potent activator of the ClpP-induced integrated stress response, we investigated the role of cellular stress in ONC201’s anti-proliferative and anti-invasive effects. The OVCAR5 and SKOV3 cells were treated with vehicle control or ONC201 for 72 hours in the presence and absence of 1 mM of the antioxidant, N-acetylcysteine (NAC). The results showed that NAC partially reversed the cytotoxic effects of ONC201 in both cell lines compared to the control cells (**Figures 7A, B**, p<0.05). Similarly, pre-treatment of NAC for 6 hours effectively reversed ONC201-induced decreases in mitochondrial membrane potential and inhibited ONC201-induced increases in intracellular ROS levels (**Figures 7B, C**, p<0.05).

Because NAC significantly inhibited ONC201-induced oxidative stress, efforts were made to explore whether ONC201 exerted anti-invasive effects through oxidative stress pathways. The wound healing assay was used to detect the ability of invasion after ONC201 treatment in both cell lines. In the presence of 10 μM or 100 μM ONC201, pre-treatment with NAC for 6 hours partially prevented ONC201-induced anti-invasive activity by 43.5% and 35.0% in the OVCAR5 cells, and 29.2% and 36.2% in the SKOV3 cells, respectively (**Figure 7D**, p<0.01). Western blotting results indicated that 1 mM NAC treatment did not change the expression of CLpP induced by ONC201. However, in NAC-treated groups, NAC partially blocked 10 μM ONC201-evoked decreases in VEGF and Snail expression (**Figure 7E**). Therefore, it appears that ONC201 exerted its anti-proliferative and anti-invasive effects partially through the integrated stress response in OC cells.

### ONC201 Inhibited P13K/AKT/mTOR and MAPK Pathways *In Vitro* and *In Vivo*

**Figure 8:**
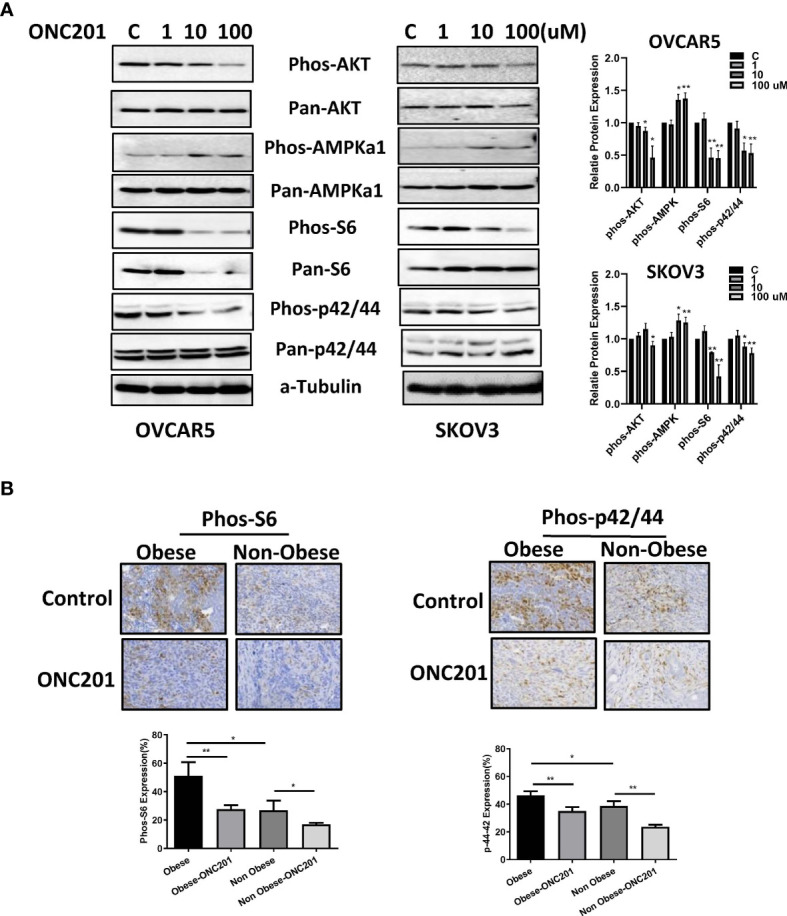
ONC201 activated AMPK and inactivated AKT/mTOR and MAPK pathways. The OVCAR5 and SKOV3 ovarian cell lines were incubated with ONC201 at various concentrations for 24 hours. Western blotting results indicated ONC201 decreased the expression of phosphorylated Akt, S6, p42/44, and increased the phosphorylated AMPK expression in both cell lines **(A)**. The KpB mice were treated with ONC201 for 4 weeks. IHC results showed that obesity increased expression of phosphorylation of S6 and p42/44, and ONC201 significantly decreased the expression of phosphorylated p-S6 and p42/44 in ovarian tumors under obese and lean conditions **(B)**. * <0.05, ** <0.01.

To gain insight into the role of ONC201 in TRAIL-mediated signaling, we evaluated whether P13K/AKT/mTOR and MAPK pathways were involved in the anti-proliferative effects of ONC201 in OC cells. OVCAR5 and SKOV3 cells were treated with ONC201 (1, 10 and 100 uM) for 24 hours. mTOR activity was determined by phosphorylation of S6 (Ser235/236) and MAPK activation by detecting P42/44 phosphorylated on Thr202 and Tyr204. ONC201 inhibited AKT phosphorylation and activity of mTOR and MAPK pathways without changing the total levels of S6, p42/44 and AKT proteins in the both cells compared with control cells (**Figure 8A**). The effects of ONC201 on the phosphorylation-dependent activation of p42/44 and S6 in the KpB serous OC mouse model are shown in **Figure 8B**. ONC201 reduced p42/44 phosphorylation by 24.3% in obese mice and 38.8% in lean mice, respectively (p<0.05). Similarly, ONC201 also reduced phosphorylation of S6 by 46.1% and 37.3% in obese and lean mice, respectively, compared to untreated mice. Overall, these data confirm that ONC201 reduces cell growth *via* inhibition of the AKT/mTOR and MAPK signaling pathways in OC cells and tumors.

## Discussion

The heterogeneous expression of dopamine receptors in different types of cancer cells suggests that these receptors may exhibit varying functions to either stimulate or inhibit cancer cell growth (31, 32). TCGA data showed that DRD2 is elevated in several types of human cancer including OC (33, 34), and the high expression of DRD2 is often associated with a decrease in the risk of OC progression and prognosis. Interestingly, targeting DRD2 by antagonists significantly reduces the viability of cancer cells and slows tumor growth *in vivo* in OC pre-clinical models (35). ONC201 is the first selective antagonist of D2-like dopamine receptors for clinical oncology (6, 10). In this study, we investigated the impact of the anti-tumorigenic activity of ONC201 in human OC cell lines and the KpB transgenic mouse model of OC under obese and lean conditions. The significant finding of this study was that ONC201 reduced the expression of DRD2, and inhibited ovarian tumor growth and reduced the ability of invasion *via* activation of ClpP induced oxidative stress pathways and inactivation of P13K/AKT/mTOR and MAPK pathways. Additionally, ONC201 induced apoptosis and cell cycle arrest in G1 phase in OC cells. Obesity induced by a HFD significantly promoted ovarian tumor growth as compared to LFD-fed mice. ONC201 significantly reduced tumor growth in both obese and lean KpB mice, accompanied by the decrease in Ki-67 and the increase ClpP expression in tumor tissues. These results indicate that the anti-cancer activity of ONC201 exceeds its ability to antagonize DRD2, which may be due to the ability of ONC201 to activate the ClpP pathway independent of DRD2.

DRD2 Inhibition through genetic or pharmacological approaches has been shown to cause apoptosis and induce cell cycle arrest in leukemia, lung, colon, breast, endometrial, cervical, ovarian, pancreatic and brain cancer cells (11, 12, 35–40). ONC201 has recently been shown to induce apoptosis *via* targeting DRD2 in a wide variety of different cancer types. The role of ONC201 mediated apoptotic pathways in its anti-tumorigenic activity have been shown to differ, depending on the cancer type. ONC201 was originally characterized as a selective antagonist of DRD2 to induce TRAIL and DR5 pathways, independent of p53 in cancer cells (41). In desmoplastic small round cell tumors, ONC201 increased the expression of TRAIL and DR5 and cell death *via* the extrinsic pathway of apoptosis (42). A recent study reported that the cytotoxicity of ONC201 was not dependent on either TRAIL death receptors or caspase cascades in breast cancer cells (43), whereas another study showed that ONC201 induced cell death that appeared to be through TRAIL-dependent and TRAIL-independent effects in breast cancer cells (44). Similar observations were noted in our recent study where we showed that ONC201 reduced BCL-2 expression and induced DR5 *via* extrinsic and intrinsic apoptotic pathways in USC cells (11). In agreement with these studies, we found that ONC201 treatment reduced BCL-XL and MCL-1 expression, induced DR5 upregulation and increased activity of cleaved caspase 3, 8 and 9 in OC cells. These findings indicate that ONC201-induced cell death can occur by both TRAIL-dependent and a TRAIL-independent mechanisms in OC cells, suggesting that the mechanism of ONC201 induced cell cytotoxicity may involve tissue- or cancer type-specific pathways in response to ONC201 (11, 17, 41, 45).

There is considerable evidence that DRD2 deficiency causes to endoplasmic reticulum (ER) stress which is required for the control of cancer cell growth (37, 46, 47). The anti-tumorigenic effects of ONC201 appear to be reliant on disrupting mitochondrial function, including inhibition of oxidative phosphorylation and reduction of the number of viable mitochondria (43). Several studies have reported that the tumor cell sensitivity to ONC201 is dependent on the induction of DR5 in an ATF4 and CHOP-dependent manner (48–50). In a phase I trial, ONC201 triggered the ISR along with induction of CHOP and TNFRSF10B in an ibrutinib-refractory mantle cell lymphoma patient following 16 days of treatment, which aligned with therapeutic exposure to ONC201 and target engagement in the tumor (15). Importantly, Graves et al. recently applied an unbiased affinity proteomics approach and Ishizawa et al. screened a small in-house library of 747 molecules approved for clinical use for malignant indications, and both discovered that ClpP is a critical molecular target that binds ONC201 in a direct and specific manner. Knockdown of ClpP by SiRNA decreased the response to ONC201 and blocked the expression of CHOP and the cytostatic effects induced by ONC201 in breast cancer cells. Activation of ClpP by ONC201 was associated directly with its anti-tumor activity through induction of ISR *in vitro* and *in vivo* (7, 8). Here, our results revealed that ONC201 depolarized mitochondrial membranes and increased ROS levels in a dose-dependent manner, accompanied by an increase in the expression of ATF4, CHOP, PERK and Ero1-1α in OC cells, which are markers of oxidative stress related to apoptosis. We also confirmed that ONC201 treatment significantly induced the expression of ClpP in OC cells and KpB mice under obese and lean conditions. SiRNA knockdown of ClpP in OC cells reduced the inhibitory effects of ONC201 although we did not observe a complete recovery of cell inhibition induced by ONC201 in ClpP knockdown cells. Overall, these studies suggest that: (1) cellular stress contributed to the anti-tumorigenic effects of ONC201 in addition to apoptosis and G1 cell cycle arrest in OC cells (37, 51), and (2) ClpP activation is partially responsible for the anti- tumorigenic activity of ONC201, and activation of ClpP provides a targeted approach to activate ER stress in cancer cells (52).

PI3K/AKT/mTOR and MAPK/ERK pathways are responsible for mediating cell proliferation, invasion and tumorigenesis in OC. The PI3K/AKT/mTOR pathway is unregulated in approximately 70% of OC patients while activated MAPK pathway was more frequently expressed in low-grade (81%) as compared with high-grade ovarian serous carcinomas (41%) (53–55). The dysregulation of AKT and ERK signaling pathways in OC opens the possibility of actively targeting the signaling cascades, which might lead to superior anti-tumor activity. Several PI3K/AKT/mTOR inhibitors and MAPK/ERK targeted therapies are currently under evaluation in clinical trials against a variety of human cancers, including OC (56). In the current study, we found that ONC201 simultaneously reduced phosphorylation of AKT, S6 and p42/44 in OC cells *in vitro*. ONC201 strongly decreased the expression of phosphorylated S6 and p42/44 in the obese and lean KpB mice. The inhibition of PIK3CA/AKT/mTOR and MAPK pathways by ONC201 could be one of the major mechanisms by which it inhibited overall OC growth in the KpB mouse model.

Peritoneal dissemination and local invasion by OC cells are involved in early steps of the metastatic process, which includes the transcriptional activation of ZEB1, TWIST, Slug and Snail, upregulation of E-cadherin and acquisition of a unique expression profile of EMT (57). The profile of EMT has been considered as a key hallmark for adhesion and invasion, and inhibition of EMT-related processes makes it particularly attractive for treatment of cancer (58). The regulatory role of DRD2 in adhesion and invasion has not been fully characterized, and the results remain controversial. Overexpression of DRD2 in patients with gastric cancer had shorter survival durations, and targeting DRD2 by thioridazine significantly inhibited cell proliferation in gastric cancer and reduced cell migration *via* suppression of EMT related genes in liver cancer (33, 59). However, increased expression of DRD2 in a neuroendocrine tumor patient was associated with longer survival, and activation of DRD2 by the DRD2 agonist BIM53097 reduced the ability of migration and invasion of human tumorous pituitary cells (34, 60). Similarly, fisetin as a DRD2 agonist suppressed liver cancer cell proliferation and reduced EMT through VEGFR1, p-ERK1/2, p38 and pJNK pathways (61). Recently, Wagner et al. found that ONC201 is able to inhibit cancer cell invasion and exhibits a potent anti-metastatic effect in a TRAIL-dependent manner (62). Our previous work also confirmed that ONC201 inhibited adhesion and invasion in USC cells, along with increasing the expression of E-cadherin and decreasing VEGF, N-cadherin and Snail expression (11). In this current study, our results are consistent with our previous proposed mechanism of action of ONC201 involving EMT processes leading to inhibition of invasion in OC cells. We also demonstrated that ONC201 reduced the invasion index in organotypic 3D cultures as well as VEGF production in serum and ovarian tumors in the KpB mice. Moreover, pretreatment with NAC reversed ONC201-decreased VEGF and Snail levels, suggesting that ClpP or ClpP-induced oxidative stress may trigger the processes of adhesion and invasion induced by ONC201 in OC cells. These studies have provided insights into the mechanisms of the anti- metastatic effects of ONC201, which are dependent on oxidative stress.

## Conclusion

**Figure 9:**
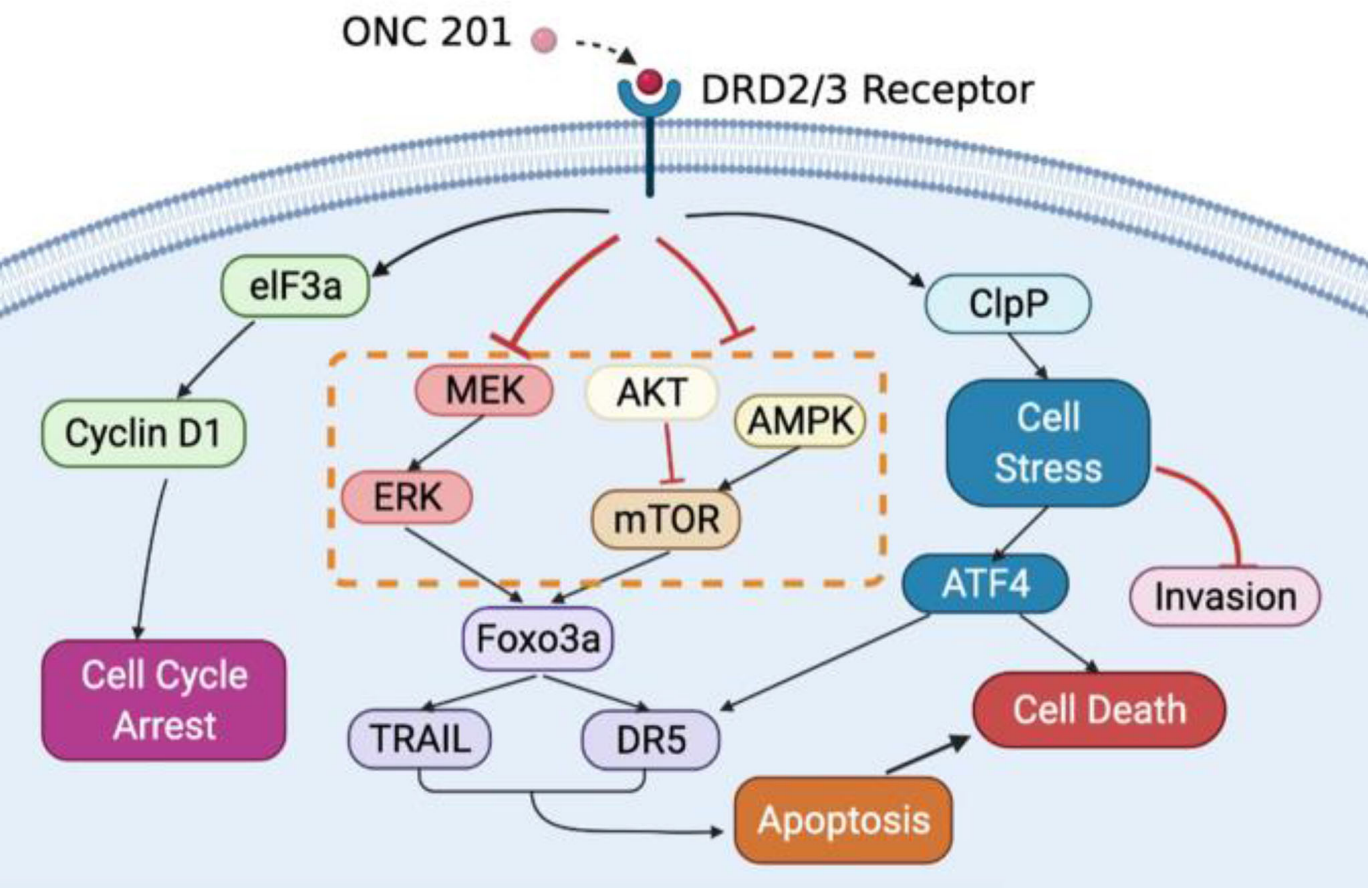
Schematic representation of the potential antitumor mechanisms of ONC201 in OC. ONC201 applies its cell growth-inhibitory impacts through DRD2/3 antagonism and ClpP activation. The connection among DRD2/3 antagonism, activation of ClpP and downstream events elicited by ONC201 including inactivation of AKT/ERK pathways, activation of the integrated stress response, induction of apoptosis, cell cycle arrest, and inhibition of invasion is summarized.

Our study uncovered for the potential anti-proliferative anti-metastatic roles of ONC201 in OC. ONC201 inhibited OC cell proliferation and tumor growth, which was associated with changes in expression of a constellation of proteins involved in apoptosis, cell cyclin, oxidative stress, angiogenesis, invasion, AMPK/mTOR and MAPK pathways (**Figure 9**). The mechanism of action of ONC201 to inhibit invasion is reliant on ClpP-mediated oxidative stress in OC. Our results have important preclinical implications indicating that ONC201 may be a promising agent in future OC clinical trial. To date, ONC201 has entered multiple clinical trials in solid tumors and hematological malignancies (63–65). However, the main limitations of our study include 100 μM of ONC21 used in the experiments being greater than the Cmax of ONC201 in the human body, knockdown experiments using a single siRNA against ClpP, and some cell line specific differences in protein expression with a high concentration of ONC201. Therefore, further research and clinical trials are needed to investigate the role of ClpP-mediated anti-proliferative and anti-metastatic effects in EMT and angiogenic pathways, and evaluate ClpP as a potential clinically useful biomarker in clinical trials of ONC201 (52).

## Data Availability Statement

The raw data supporting the conclusions of this article will be made available by the authors, without undue reservation.

## Ethics Statement

The animal study was reviewed and approved by UNC-CH Institutional Animal Care and Use Committee (IACUC). Written informed consent was obtained from the owners for the participation of their animals in this study.

## Author Contributions

CZ and VB-J conceived and designed the experiments. YF, JW, ZF, SP, LW, AS, KT, YY and WK performed the main experiments and analyzed the data. YF, ZF, and WS performed experiments *in vivo*. VP and JA provided ONC201. CZ and VB-J wrote the manuscript. All authors have read and approved the final manuscript.

## Funding

This work is supported by: (1) VB-J: American Cancer Society (AS) Research Scholar Grant - RSG CCE 128826. (2) VB-J: NIH/NCI - R37CA226969. (3) JW: Beijing health system high-level health personnel training program fund (2014-3-073).

## Conflict of Interest

VP and JA are employees and stockholders of Oncoceutics.

The remaining authors declare that the research was conducted in the absence of any commercial or financial relationships that could be construed as a potential conflict of interest.

## Publisher’s Note

All claims expressed in this article are solely those of the authors and do not necessarily represent those of their affiliated organizations, or those of the publisher, the editors and the reviewers. Any product that may be evaluated in this article, or claim that may be made by its manufacturer, is not guaranteed or endorsed by the publisher.
