# Supplementary material for: Anti-Tumor and Anti-Invasive Effects of ONC201 on Ovarian Cancer Cells and a Transgenic Mouse Model of Serous Ovarian Cancer
Source: Front Oncol. 2022 Mar 17;12:789450. doi: 10.3389/fonc.2022.789450 (PMC8970020; doi:10.3389/fonc.2022.789450)
Supplement: Supplementary file 1 [file DataSheet_1.pdf]

Fig 1.

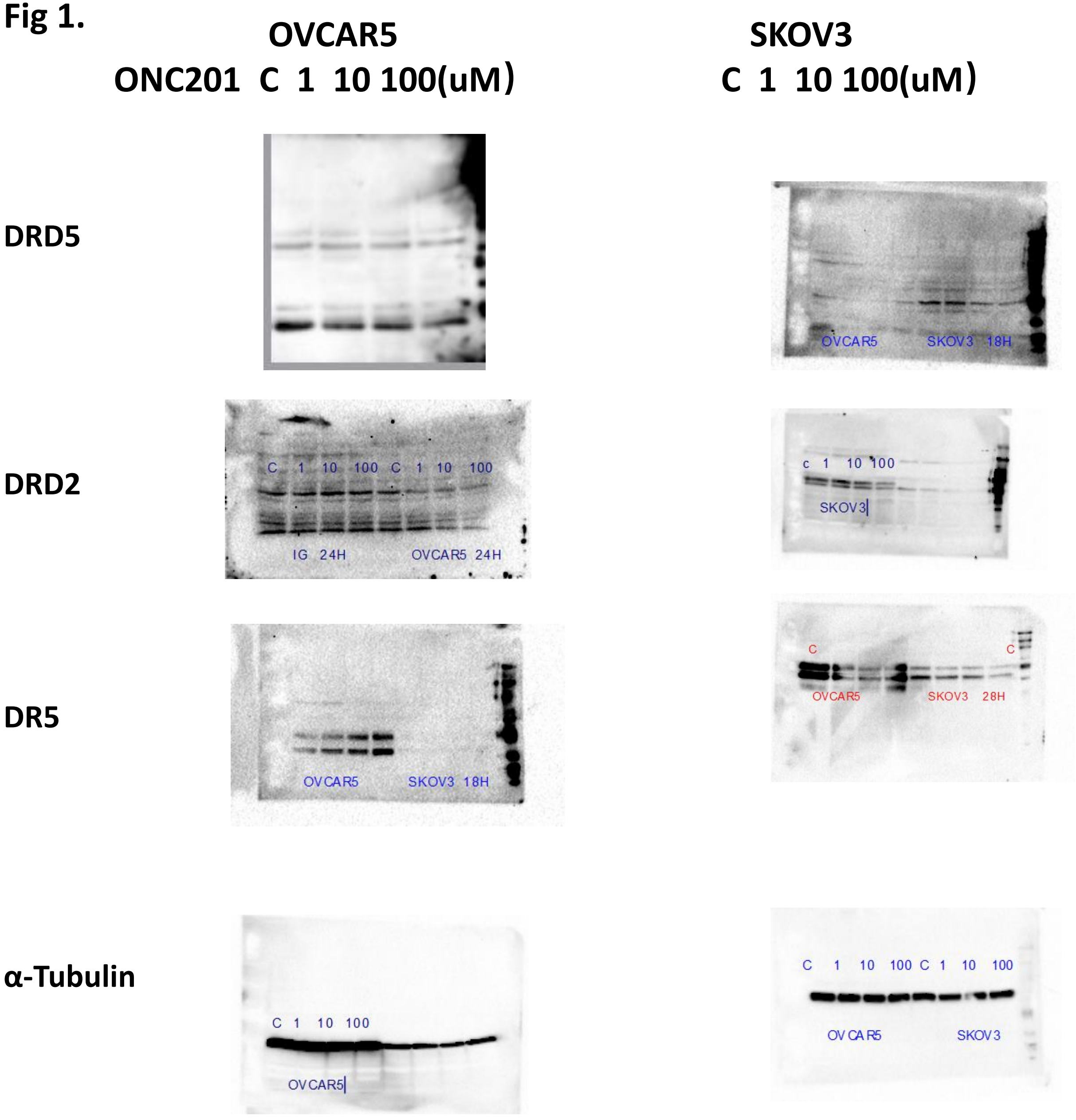
OVCAR5
SKOV3
ONC201 C 1 10 100(uM)
C 1 10 100(uM)
DRD5
OVCAR5
SKOV3 18H
DRD2
C 1 10 100 C 1 10 100
IG 24H
OVCAR5 24H
c 1 10 100
SKOV3
DR5
OVCAR5
SKOV3 18H
C
C
OVCAR5
SKOV3 28H
α-Tubulin
C 1 10 100
OVCAR5
C 1 10 100 C 1 10 100
OVCAR5
SKOV3

Fig 2.

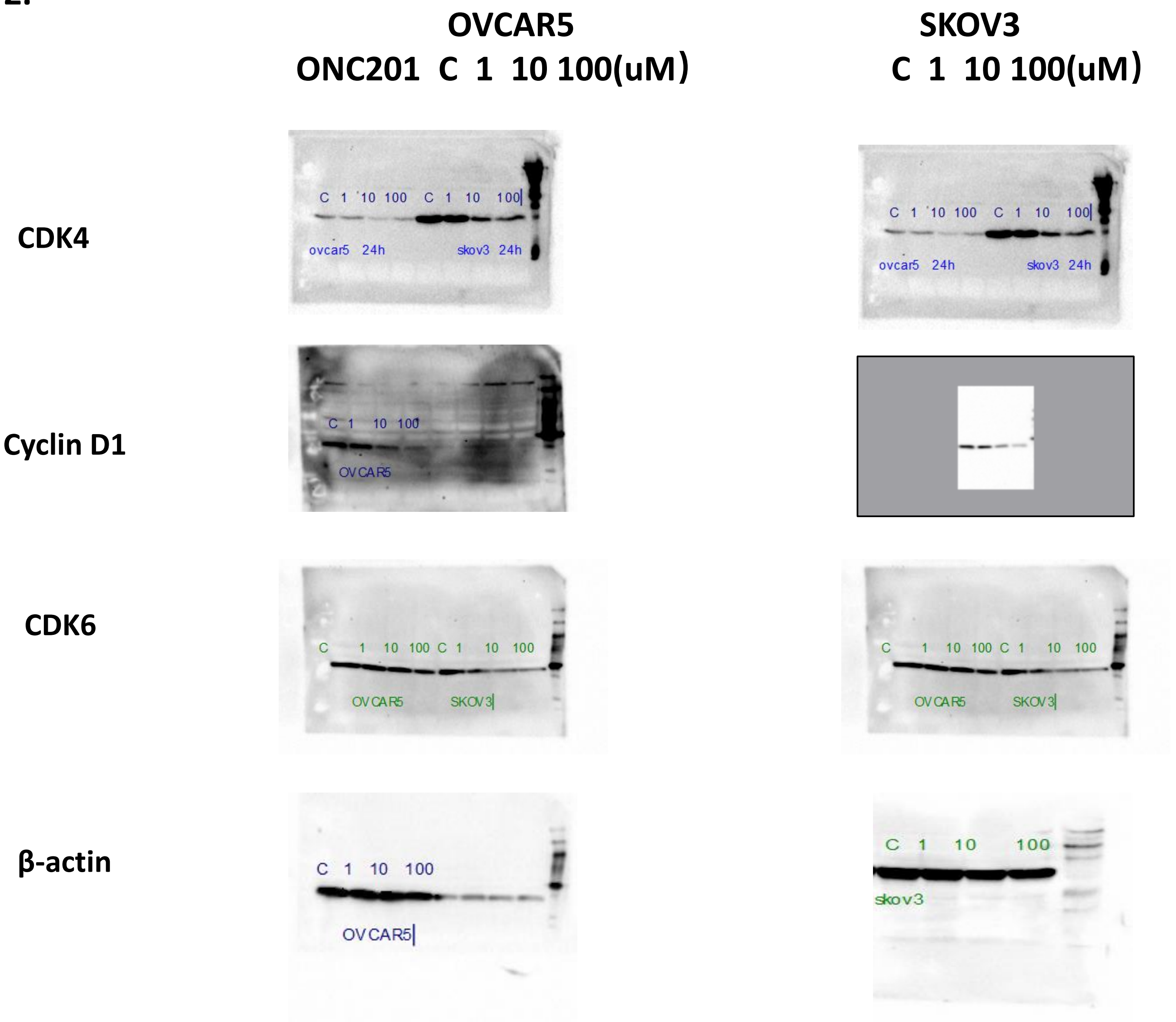
OVCAR5
ONC201 C 1 10 100(uM)
SKOV3
C 1 10 100(uM)
CDK4
Cyclin D1
CDK6
β-actin
C 1 10 100 C 1 10 100
ovcar5 24h
skov3 24h
OVCAR5
SKOV3
skov3

Fig 3.

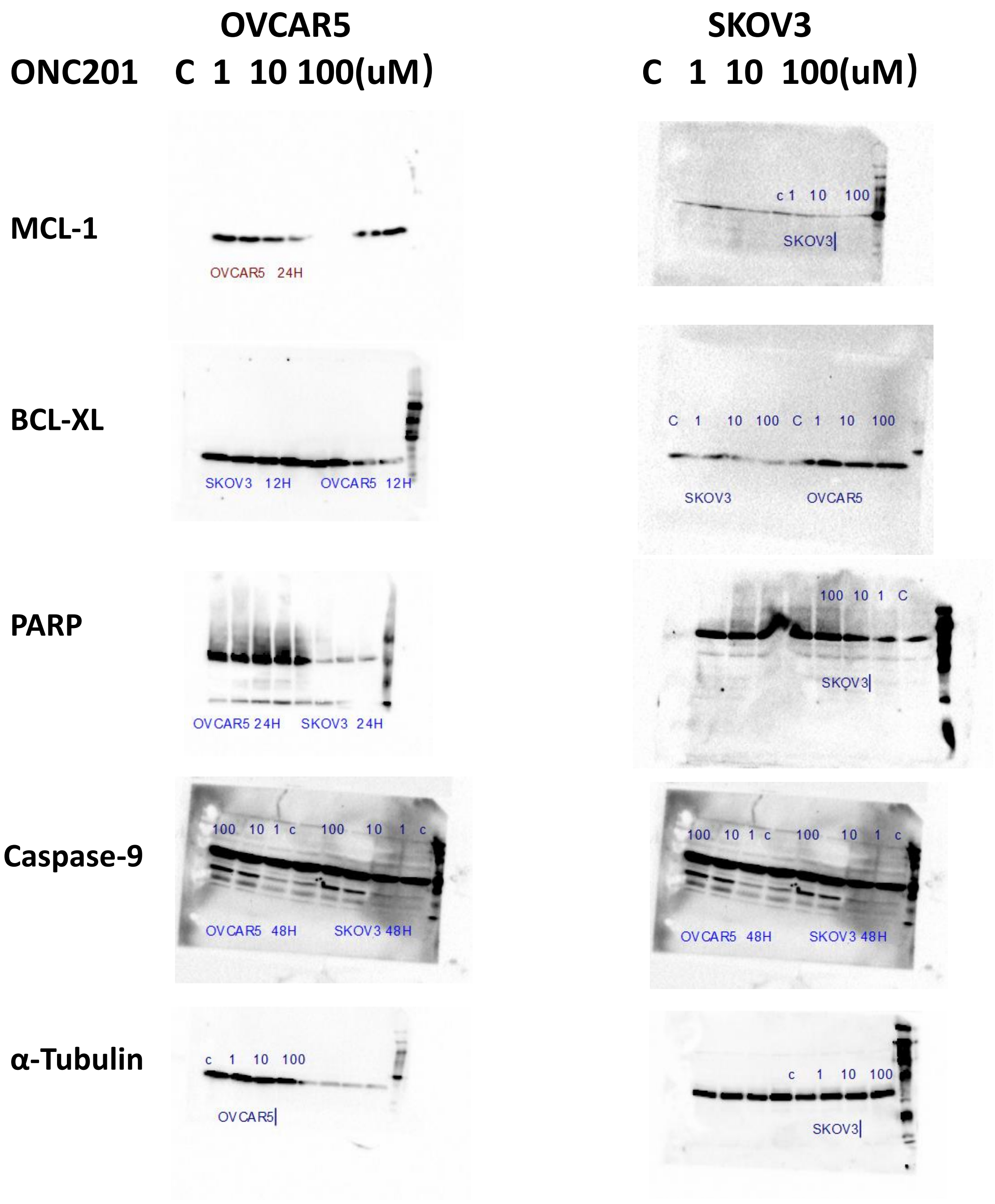
OVCAR5
SKOV3
ONC201
C 1 10 100(uM)
C 1 10 100(uM)
MCL-1
BCL-XL
PARP
Caspase-9
α-Tubulin
OVCAR5 24H
c 1 10 100
SKOV3
SKOV3 12H
OVCAR5 12H
C 1 10 100 C 1 10 100
SKOV3
OVCAR5
OVCAR5 24H
SKOV3 24H
100 10 1 C
SKOV3
100 10 1 c 100 10 1 c
OVCAR5 48H
SKOV3 48H
100 10 1 c 100 10 1 c
OVCAR5 48H
SKOV3 48H
c 1 10 100
OVCAR5
c 1 10 100
SKOV3

Fig 5.

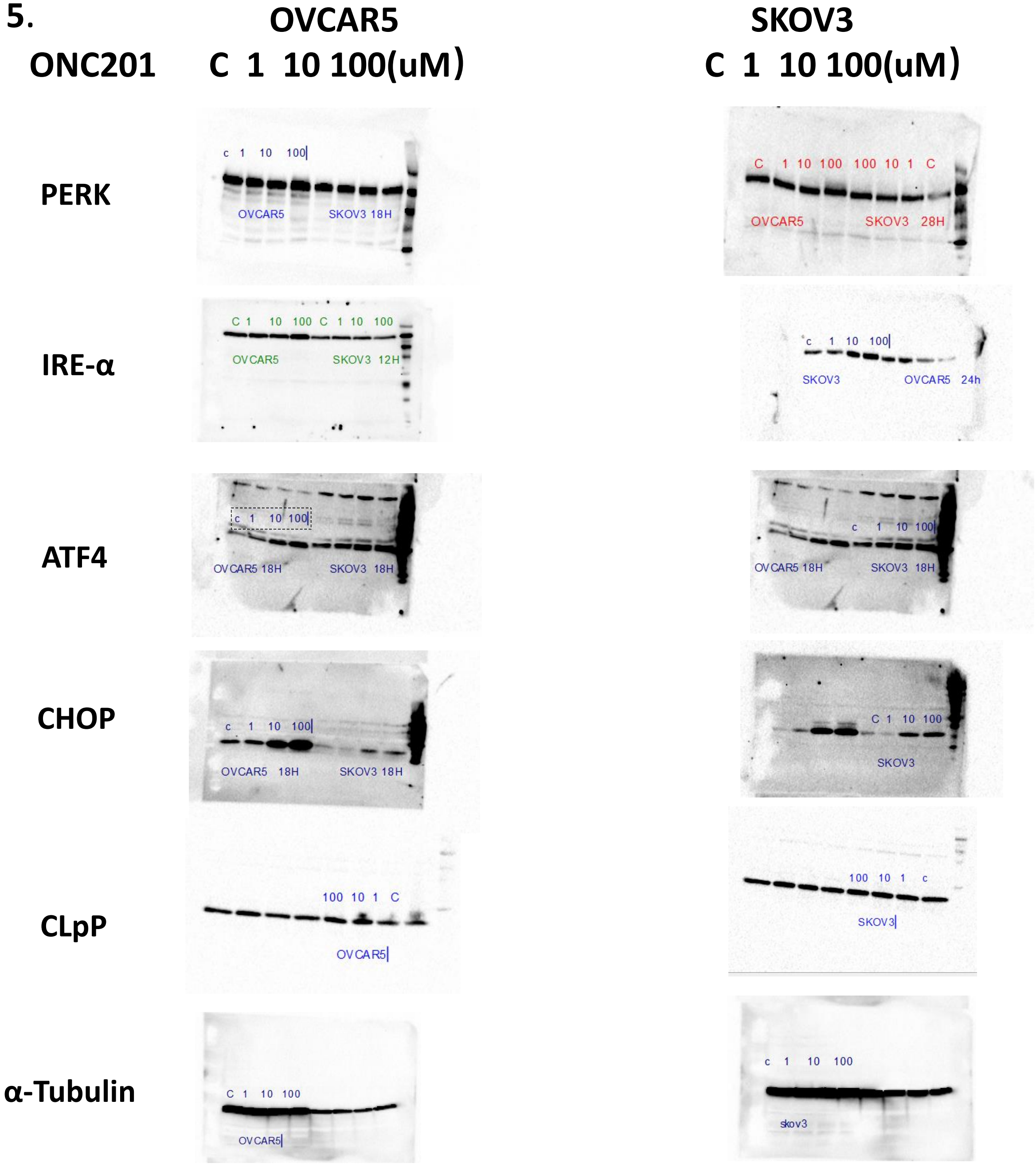
OVCAR5
SKOV3
ONC201
C 1 10 100(uM)
C 1 10 100(uM)
PERK
IRE-α
ATF4
CHOP
CLpP
α-Tubulin
c 1 10 100
OVCAR5
SKOV3 18H
C 1 10 100 100 10 1 C
OVCAR5
SKOV3 28H
C 1 10 100 C 1 10 100
OVCAR5
SKOV3 12H
c 1 10 100
SKOV3
OVCAR5 24h
c 1 10 100
OVCAR5 18H
SKOV3 18H
c 1 10 100
OVCAR5 18H
SKOV3 18H
c 1 10 100
OVCAR5 18H
SKOV3 18H
C 1 10 100
SKOV3
100 10 1 C
OVCAR5
100 10 1 c
SKOV3
C 1 10 100
OVCAR5
c 1 10 100
skov3

Fig 6.

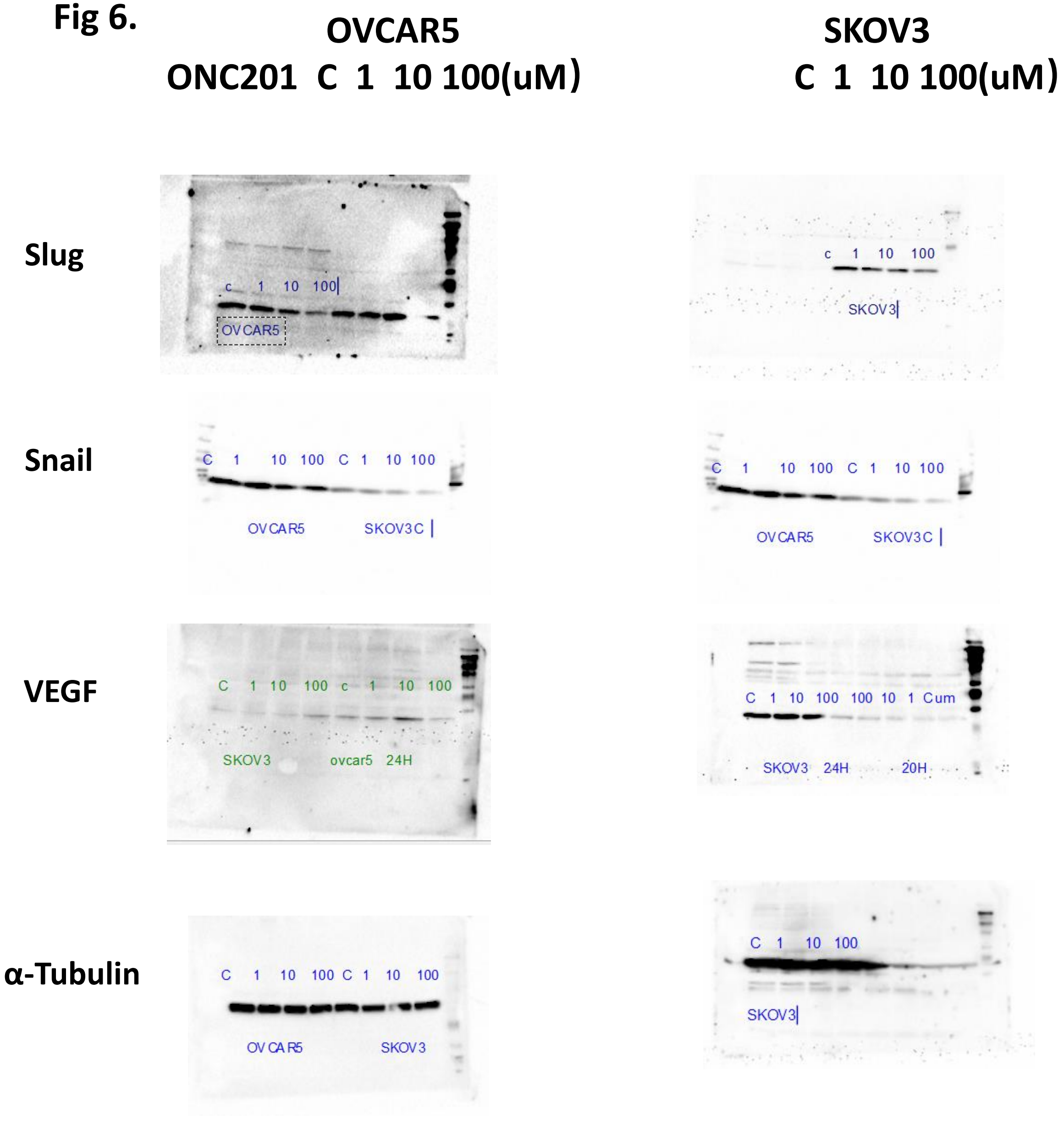
OVCAR5
ONC201 C 1 10 100(uM)
SKOV3
C 1 10 100(uM)
Slug
c 1 10 100
OVCAR5
c 1 10 100
SKOV3
Snail
C 1 10 100 C 1 10 100
OVCAR5 SKOV3C
VEGF
C 1 10 100 c 1 10 100
SKOV3 ovcar5 24H
C 1 10 100 100 10 1 Cum
SKOV3 24H 20H
α-Tubulin
C 1 10 100 C 1 10 100
OVCAR5 SKOV3
C 1 10 100
SKOV3

Fig 7.

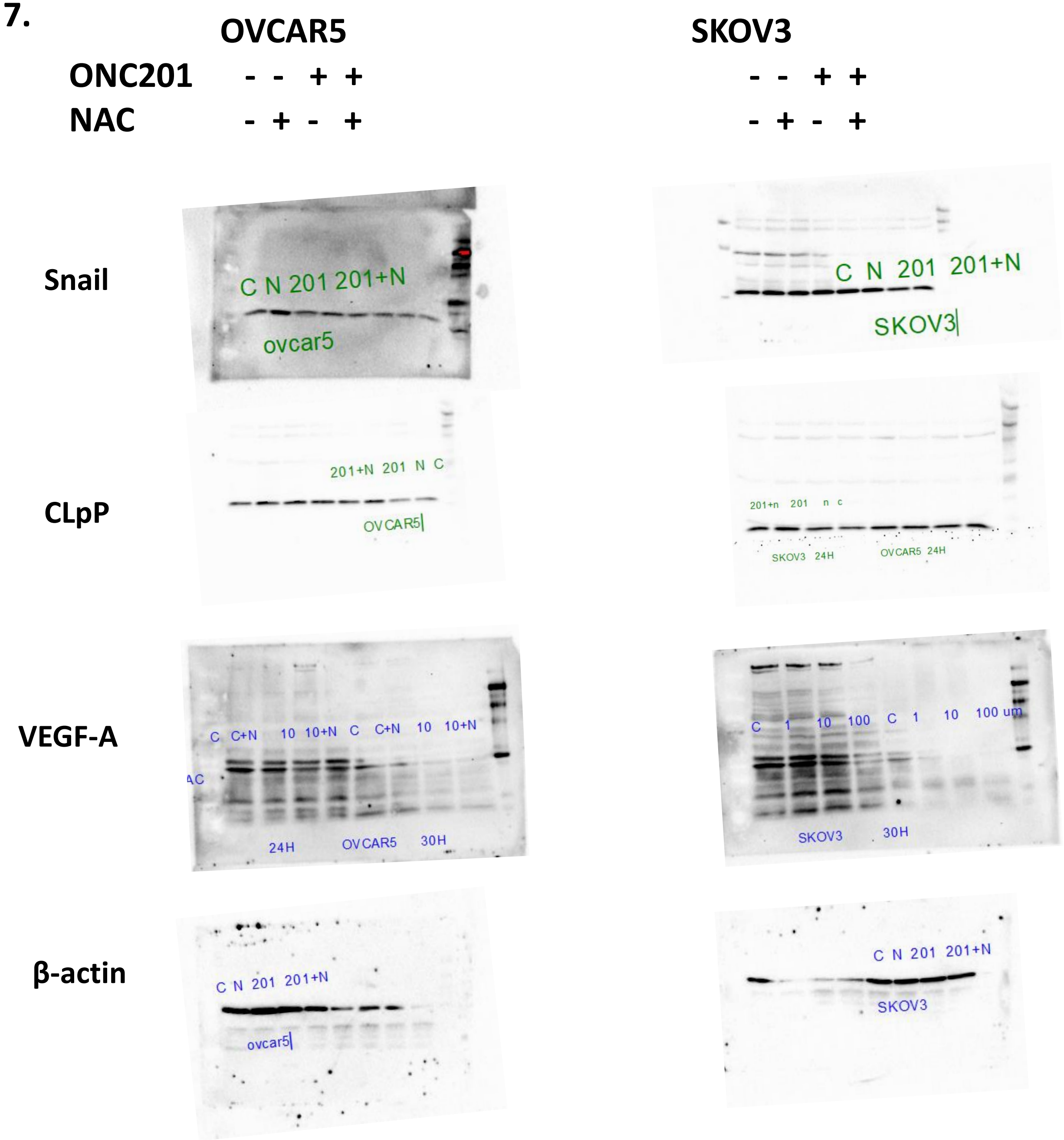
OVCAR5
SKOV3
ONC201
NAC
- - + +
- + - +
- - + +
- + - +
Snail
CLpP
VEGF-A
β-actin
C N 201 201+N
ovcar5
C N 201 201+N
SKOV3
201+N 201 N C
OVCAR5
201+n 201 n c
SKOV3 24H
OVCAR5 24H
C C+N 10 10+N C C+N 10 10+N
24H
OVCAR5
30H
C 1 10 100 C 1 10 100 um
SKOV3
30H
C N 201 201+N
ovcar5
C N 201 201+N
SKOV3

Fig 8.

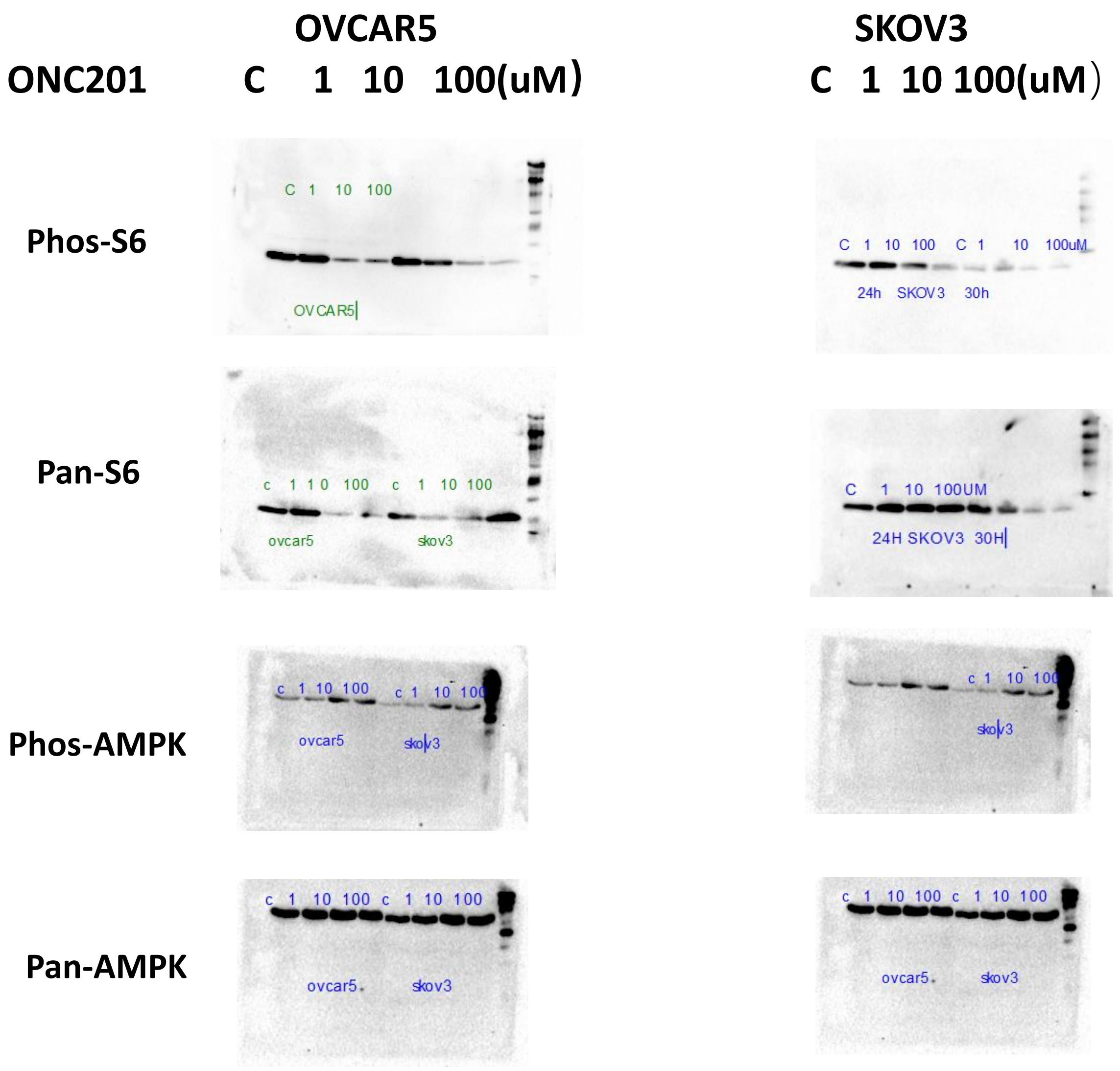
OVCAR5
SKOV3
ONC201
C 1 10 100(uM)
C 1 10 100(uM)
Phos-S6
Pan-S6
Phos-AMPK
Pan-AMPK
C 1 10 100
OVCAR5
C 1 10 100 C 1 10 100uM
24h SKOV3 30h
c 1 10 100 c 1 10 100
ovcar5
skov3
C 1 10 100UM
24H SKOV3 30H
c 1 10 100 c 1 10 100
ovcar5
skov3
c 1 10 100
skov3
c 1 10 100 c 1 10 100
ovcar5
skov3
c 1 10 100 c 1 10 100
ovcar5
skov3

OVCAR5 SKOV3

ONC201 C 1 10 100(uM) C 1 10 100(uM)

Phos-AKT

Pan-AKT

α-Tubulin

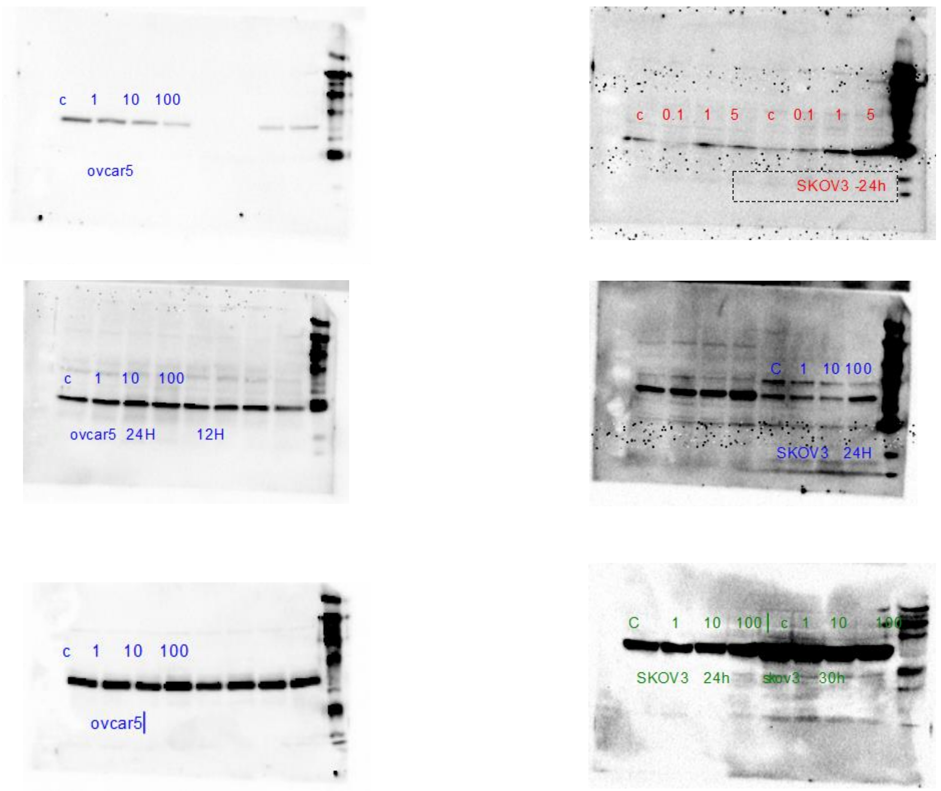

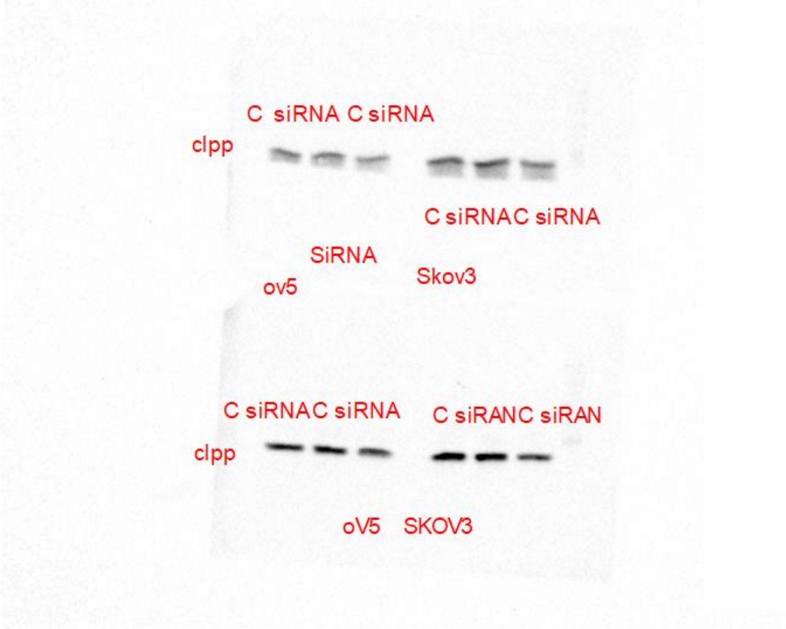
C siRNA C siRNA
clpp
C siRNAC siRNA
SiRNA
ov5
Skov3
C siRNAC siRNA
C siRANC siRAN
clpp
oV5
SKOV3

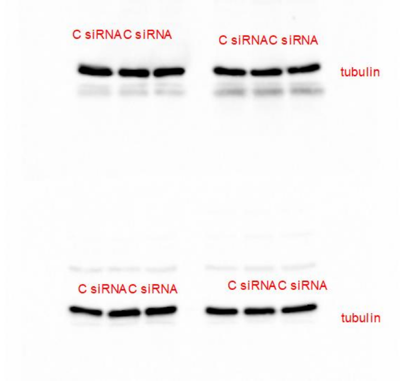
C siRNAC siRNA
C siRNAC siRNA
tubulin
C siRNAC siRNA
C siRNAC siRNA
tubulin

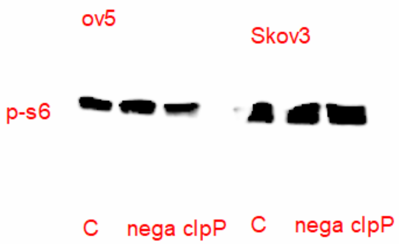
ov5
Skov3
p-s6
C
nega clpP
C
nega clpP

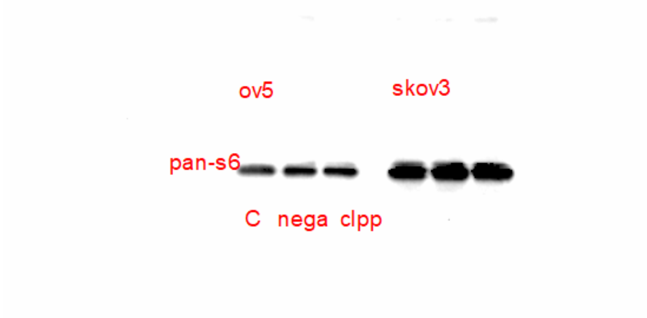
ov5
skov3
pan-s6
C
nega
clpp

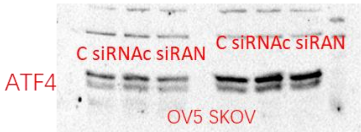
ATF4
C siRNAc siRAN
C siRNAc siRAN
OV5 SKOV

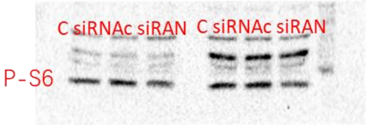
C siRNAc siRAN
C siRNAc siRAN
P-S6

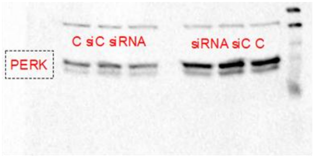
C siC siRNA
siRNA siC C
PERK

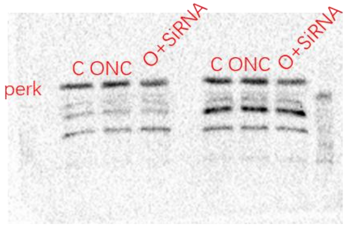
C ONC
O+SiRNA
C ONC
O+SiRNA
perk
